# *Portunus pelagicus* (Linnaeus, 1758) as a Sentinel Species to Assess Trace Metal Occurrence: A Case Study of Kuwait Waters (Northwestern Arabian Gulf)

**DOI:** 10.3390/toxics11050426

**Published:** 2023-05-03

**Authors:** Qusaie Karam, Wassim Guermazi, M. N. V. Subrahmanyam, Yousef Al-Enezi, Mohammad Ali, Vincent Leignel, Neila Annabi-Trabelsi

**Affiliations:** 1 Environment and Life Sciences Research Center, Kuwait Institute for Scientific Research, P.O. Box 24885, Kuwait City 13109, Kuwait; qkaram@kisr.edu.kw (Q.K.); yaenezi@kisr.edu.kw (Y.A.-E.); mohalikisr@gmail.com (M.A.); 2 Université de Sfax, Biodiversité Marine et Environnement (LR18ES30), Route Soukra Km 3.5, B.P. 1171, Sfax 3000, Tunisia; wassim.guermazi@fss.usf.tn; 3 Department of Biological Sciences, Faculty of Science, Kuwait University, P.O. Box 5969, Kuwait City 13060, Kuwait; drsubra@hotmail.com; 4 Laboratoire BIOSSE, Le Mans Université, 72000 Avenue O Messiaen, France; vincent.leignel@univ-lemans.fr

**Keywords:** *Portunus pelagicus*, sentinel, seawater, sediments, heavy metals, bioaccumulation, Kuwait

## Abstract

Heavy metal pollution can adversely impact marine life, such as crabs, which can accumulate it in different organs and potentially transfer and biomagnify along the food chain in aquatic ecosystems. This study aimed to examine the concentrations of heavy metals (Cd, Cu, Pb, and Zn) in sediment, water, and crab tissues (gills, hepatopancreas, and carapace) of the blue swimmer crab *Portunus pelagicus* in the coastal areas of Kuwait, northwestern Arabian Gulf. Samples were collected from Shuwaikh Port, Shuaiba Port, and Al-Khiran areas. The accumulation of metals in crabs were higher in the carapace > gill > digestive gland, and the highest metal concentration was found in crabs collected from Shuwaikh > Shuaiba > Al-Khiran. The metal concentrations in the sediments were in the order Zn > Cu > Pb > Cd. Zn was the highest metal concentration detected in marine water sampled from the Al-Khiran Area, whereas the lowest metal was Cd sampled in water from the Shuwaikh Area. The results of this study validate the marine crab *P. pelagicus* as a relevant sentinel and prospective bioindicator for evaluating heavy metal pollution in marine ecosystems.

## 1. Introduction

*Portunus pelagicus* (Linnaeus, 1758), commonly known as the blue swimmer crab or sand crab, is a species of economic importance in many parts of the world [1]. Thus, this species is an important component of small-scale coastal fisheries worldwide [2,3,4]. For example, *P. pelagicus* is in demand in the markets of distinct Asian countries such as Thailand, Malaysia, and China [5].

*P. pelagicus* has a key role in the marine ecosystem because it is not only a predator, but also a scavenger and a cannibal [6]. Bivalves, crustaceans, gastropods, fish, and ophiuroids are the dominant components of its diet, but these animals are able to ingest any food item depending on the availability [7,8]. This species appears to be a common member of the rich intertidal fauna of Kuwait that dominates the sublittoral zone of the coastal area as a fast-swimming predator [9,10,11]. In parallel, it is considered a commercial seafood product by both locals and expatriates. Blue swimmer crab total landing in Kuwait has not been documented; however, since Kuwait shares the same region as other Arabian Gulf countries, the total landings were 3248 tons in Bahrain and 4472 tons in Saudi Arabia [8,12].

## 2. *Portunus pelagicus*: Description, Phylogeny, Distribution and Habitat, Life Cycle, and Physiological Capacities

### 2.1. Description of Portunus pelagicus

**Figure 1:**
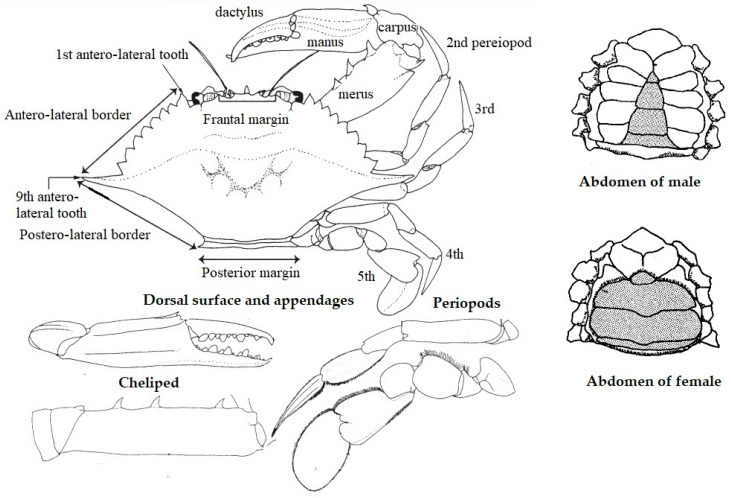
Schematic drawings of a generalized *Portunus pelagicus* illustrating morphology. (Lai et al. [14] modified).

First described by Carl Linnaeus in 1758 as *Cancer pelagicus*, this species was later transferred to *Portunus* (Weber, 1795) and included in Portunidae [13]. As implicit from its name, the blue swimmer crab is a distinguished swimmer, basically because of the modification of its last pair of walking legs. It has five pairs of walking legs (Figure 1). The first pair was altered into a long, skinny cheliped with a combination of the merus, carpus, manus, and dactylus, and three sharp spines on the inner margins of the merus. The second, third, and fourth walking legs are slender and lengthened. The fifth pair is oval, paddle-shaped at the end, and rotates easily to help swimming in all directions [14]. This modification of the legs to facilitate swimming can be observed in other genera, including Portunidae, such as *Callinectes*, *Liocarcinus*, *Scylla*, and *Thalamita*.

The carapaces are greenish-brown in color, with irregular, pale mottling and dark brown edges. Chelipeds are purplish and mottled, and their fingers are blue. The carapace is broad in breadth with transverse granulate lines. The breadth–length ratio is 2/13. The front edge has four acutely triangular teeth, with nine teeth on each anterolateral margin (including acute lateral orbital angle teeth). The last pair of teeth is 2–4 times larger than the preceding teeth. Chelipeds are long, massive, spinous, and ridged. The inner margin of the merus (cheliped) has three spines.

This species displays sexual dimorphism. Females are better camouflaged in a uniform dull green-brown carapace with red-tipped chelipeds. Males acquire a dark blue-green carapace with purple-blue, red-tipped chelipeds. The abdomen shape also differs between sexes (Figure 1). The abdomens of males are narrow, pointed, and rostrally. Females are characterized by broad triangle-shaped abdomens for carrying eggs during breeding [15].

### 2.2. Phylogeny

**Figure 2:**
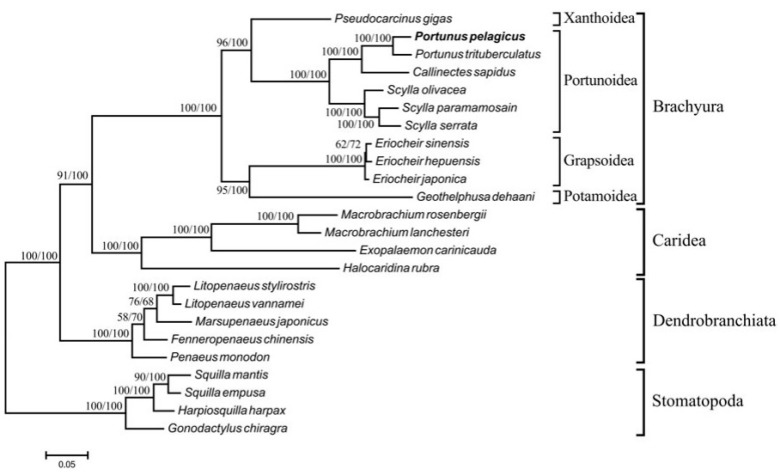
Phylogenetic tree of decapod relationships from the dataset of 13 concatenated mitochondrial PCGs. Four stomatopods served as outgroups [16].

Four complete mitogenome sequence records of this species, KT382858.1, KR153996.1, NC_026209.1, and KM977882.1, are available in GenBank. The mitogenome of *P. pelagicus* contains the same set of genes and one control region as the other metazoans (Figure 2). *P. pelagicus* is logically included in the Portunoidea [13] superfamily.

**Figure 3:**
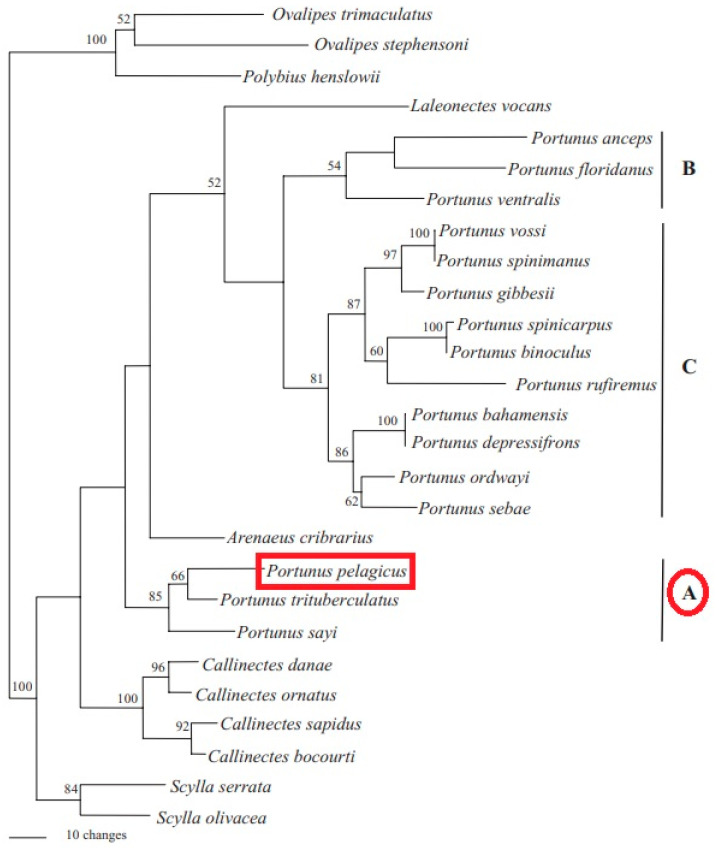
Consensus phylogenetic tree of 16S rRNA gene sequences by [17]. Numbers on the tree are bootstrap values. A, B, and C are the 3 major clades.

More precisely, mitochondrial DNA (mtDNA) was used to determine the phylogenetic relationship between distinct species of the genus *Portunus*. Phylogenetic analyses mined using 16S sequences showed that the genus *Portunus* was divided into three lineages (clades A, B, and C; Figure 3) [17]. Clade (A) shared a common basal lineage with *Arenaeus* and *Callinectes* in the analysis, whereas clades B and C were derived from an independent common lineage.

The type species of *P. pelagicus*, *Portunus trituberculatus* (Miers, 1876), and *P. sayi* (Gibbes, 1850) were clustered together.

**Figure 4:**
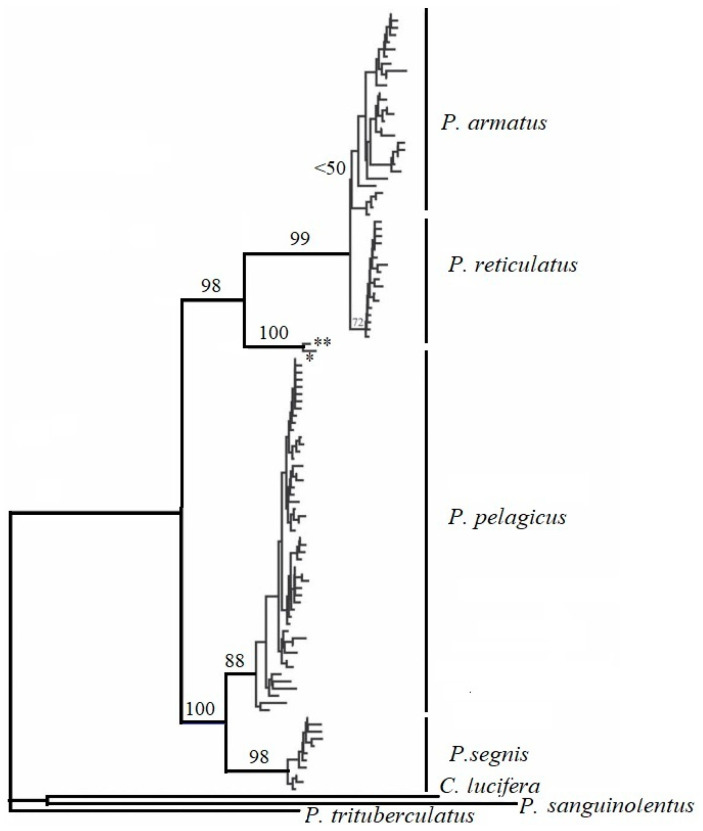
Minimum evolution bootstrap tree including all unique COI haplotypes by [14] ‘*’ Indicates the dominant haplotype found in *P. pelagicus* shared with eight *P. reticulatus* individuals. ‘**’ Denotes two individuals collected from Japan that may constitute a possible cryptic species.

A more recent phylogenetic study (using mitochondrial COI) revealed that *P. pelagicus* is in fact a species complex. Genetic data separated “*P. pelagicus*” into four sister species: *P. armatus* (A. Milne-Edwards, 1861), *P. pelagicus*, *P. reticulatus* (Herbst, 1799), and *P. segnis* (Forskål, 1775) (Figure 4). According to [14], *P. pelagicus* sensu stricto is extensive across Southeast and East Asia and is sympatric with *P. armatus* in northern Australia. *P. armatus* is observed around most of Australia and east of New Caledonia. *P. reticulatus* exists in the eastern Indian Ocean, and it is confirmed that the Bay of Bengal is a zone of hybridization for *P. pelagicus* and *P. reticulatus* [14]. *P. segnis* seems to be enclosed in the western Indian Ocean from Pakistan to South Africa and is a Lessepsian migrant into the Mediterranean from the Red Sea [14].

### 2.3. Distribution and Habitat

The distribution of *P. pelagicus*, one of the important representatives of decapod crustaceans, covers a wide area from the southern Mediterranean Sea, the east coast of Africa across the Indian Ocean to Japan, and the western Pacific Ocean in nearshore marine embayments and estuarine systems [18,19,20]. It is a species commonly found on the Arabian Gulf coasts [10]. The blue swimmer crab also occurs in the Mediterranean Sea as a Lessepsian species along the coast of Egypt, Lebanon, Turkey, Syria, Cyprus, and the southeastern coast of Sicily [21]. It migrated to the Mediterranean through the Suez Canal. The first sample was documented in 1898 in the Levantine Basin. Its Mediterranean migration mainly traced the Anatolian coasts and continued westwards [22]. For example, the first finding of *P. pelagicus* along the Tunisian coastline (Gulf of Gabes, southeastern Tunisia) was in October 2014 by local fishermen [23].

In general, *P. pelagicus* lives in a wide range of inshore and continental shelf habitats, including sandy, muddy, or algal and seagrass habitats, from the intertidal zone to at least 50 m depth [24,25].

### 2.4. Life Cycle

**Figure 5:**
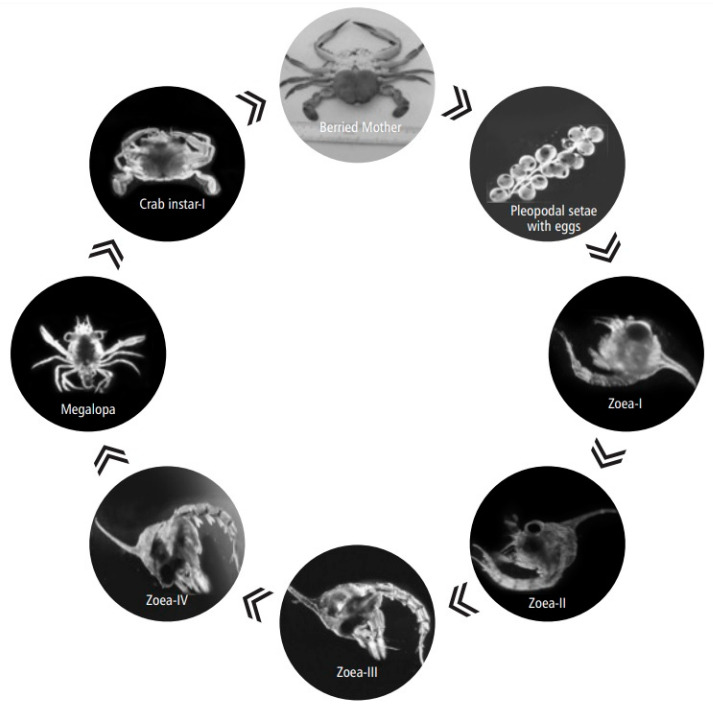
Life Cycle of *Portunus pelagicus* (Linnaeus, 1758) by [15].

Zoeal stages vary between four and eight among the family of Portuninae [26,27,28]. *Portunus pelagicus* has four zoeal stages and one megalopal stage (Figure 5). The first and second zoeal stages take 3–4 days each, the third and fourth stages take 2–3 days each, and the megalopa takes 3–4 days. The megalopa metamorphoses directly into the first crab instar. The time span of larval development is reported to be 14–17 days. The first crab instar emerges between 15 and 18 days [29]. Males are able to reach sexual maturity by their 12th molt, whereas females reach sexual maturity at their 14th molt [30].

### 2.5. Physiological Capacities

Blue swimmer crab juveniles emigrate in mass numbers preceding seasonal salinity reductions in estuaries [18]. According to [31], salinity outside of 20–35 ppt can significantly reduce the survival, development, and growth of early juvenile sand crabs. Mortality is higher for juveniles cultured at salinities ≤15 psu and at 45 psu. At a salinity of 5 psu, complete mortality occurred on day 20 [31]. The osmoregulatory ability of aquatic animals is resolved by calculating the hemolymph osmolality under different salinity conditions [32] and comparing it with the medium osmolality. *P. pelagicus* hemolymph displayed a positive linear relationship with the osmolality of the medium [31].

Critical thermal maxima (CTmax) and minima (CTmin) were determined for *P. pelagicus* acclimated at 15, 20, 24, 25, 28, 30, 32, and 35 °C [33,34]. The CTmax ranged from 38.17 °C to 44.38 °C, while the CTmin ranged from 12.28 °C to 19.30 °C [33,34], and both increased directly with temperature [34]. The high critical thermal maximum indicates the potential of this species to adapt to a wide range of temperatures and justifies its consideration as an invasive alien species. Heat tolerance plays an important role in the successful invasion of aquatic invertebrates [35,36]. The acclimation response ratio ranged from 0.25 to 0.51 [34] and from 0.05 to 0.28 [33]. The juvenile and larval stages of *P. pelagicus* are reported to be vulnerable at temperatures below approximately 17 °C [37]. Some authors [38] have described fluctuations in hemolymph serotonin levels upon thermal stress in *P. pelagicus*.

Low- and high-temperature treatments limit the locomotion of crabs [39]. Changes in temperature significantly influence the mobility of crabs in their natural habitats. An increase in water temperature from 36 °C to 20 °C can lead to a difference of 29 movements per 30 min for the blue swimmer crab [34]. No significant differences were observed in the amount of food consumed by male and female *P. pelagicus* within the temperature range 16.5–26 °C [40].

## 3. *Portunus pelagicus* as a Sentinel Species (Quantitative Bioindicator)

A sentinel organism has to fulfill some basic criteria that mainly include ease of sampling, a temporal and spatial abundance of the organism, and the range in which biological responses can be detected [41]. Crustacean species are usually considered sentinels for the estimation of toxicological impacts [42,43,44], mainly due to their high ecological plasticity, ability to alter their physiological and biochemical functions to survive in the presence of pollutants, and their wide distribution [45,46]. In addition, crustaceans that live near the bottom of water bodies have restricted mobility and are therefore powerful indicators of ecological conditions [47]. Decapod crustaceans have been suggested as potentially useful environmental indicators for coastal marine environments [48,49]. A variety of indicator types were identified as relevant for application to decapod crustaceans, such as molecular biomarker synthesis [50,51,52], bioaccumulation of toxicants [53,54], sex ratio [55], abundance [56,57], biomass [56,58], morphometrics [59,60], and prevalence of disease [61,62]. To assess the concentrations of chemical pollutants and the current status of marine ecosystems, different species of portunids can be used as sentinel organisms [63,64,65,66,67].

*P. pelagicus* also has the potential to be utilized as a sentinel of pollution in coastal ecosystems [68] given the assessed bioaccumulative ability of trace metals and organic compounds [10,18,69,70,71] and being a scavenger organism that feeds on a wide range of materials, including debris and other bottom-dwelling animals [72,73]. Crabs have the capacity to metabolize chemical pollutants adsorbed on surface sediment particles [74]. The hepatopancreas is one of the most important organs that plays important roles in metal detoxification [75,76,77].

The concentrations of heavy metals accumulated in different organs of *P. pelagicus* from the southeast coast of India exhibited the following order of concentrations in different organs: Cu > Mn > Cd > Ni > Pb > Co > Hg = Cr = U in gills, Cu > Mn > Cd > Ni > Pb = Co > Hg > Cr = U in hepatopancreas, and Cu > Cr > Ni > Mn > Cd > Pb > Co > Hg > U in muscle [78]. The order of trace metal uptake is gills > hepatopancreas > muscle [78]. In fact, heavy metals accumulate first in the gills, and excessive uptake of heavy metals into the hemolymph leads to their successive distribution in the crab’s internal organs [79]. In general, the bioaccumulation of heavy metals in tissues and organs may damage normal physiological processes in animals [80]. Sugumar [81] detected hyperglycemic hormone in the hemolymph of *P. pelagicus* exposed to heavy metals and an elevation in carbohydrate metabolite levels. Copper exposure induces an increase in oxygen consumption and heart rate in *P. pelagicus* [82]. Environmental pollution by heavy metals (AS, Cd, Hg, and Pb) negatively affected the condition of the blue swimmer crab population structure, the growth rate, and the morphometry of the carapace size [83]. These studies later reported damage to the organs of crabs exposed to heavy metals based on the degree of maturity of the gonads. Heavy metals in polluted environments are also responsible for oxidative and metabolic stress in crabs [84]. Heavy metal bioaccumulation leads to a high synthesis of antioxidants and metabolic enzymes in crabs [84]. For example, Superoxide Dismutase and Catalase are considered the primary defenses against oxidative stress and are essential for fighting superoxide anion radicals during tissue damage [85]. With increasing concentrations of heavy metals in crabs, the activities of digestive enzymes decreased [86]. Metal accumulation in marine animal tissues is affected by sex, size, seasonal changes, and the molting cycle [87].

*P. pelagicus* has a high bioaccumulation capacity similar to other marine species recognized as bioindicators. Bivalves are commonly used to assess the levels of heavy metals in marine ecosystems [88,89,90]. According to Nour [85], *Tridacna squamosa* (Lamarck, 1819) has the highest accumulation ability for Pb, Ni, and Zn; *Chama pacifica* (Broderip, 1835) has the highest accumulation capability for Cd and Co; and *Periglypta reticulata* (Linnaeus, 1758) has the highest concentration ability for Cu. *Mytilus galloprovincialis* (Lamarck, 1819) and *Tapes decussatus* (Linnaeus, 1758) are also described as important bioaccumulators of essential and nonessential metals, and their responses depend on variations in the physicochemical parameters of water (e.g., temperature, salinity, and dissolved oxygen) [91]. Furthermore, it has been reported that the concentrations of Al, Cr, and Pb in the tissues of *M. galloprovincialis* were higher than those of the same elements in the tissues of *T. decussatus* [91]. However, the levels of Cd, Cu, Mn, Ni, and Zn in the tissues of *T. decussatus* were higher compared to their counterparts in the tissues of *M. galloprovincialis* [91]. Oysters are also known to accumulate high concentrations of Cu and Zn in their tissues [92,93]. For example, high Cu (12,000 mg/g d.w) and Zn (17,000 mg/g d.w) levels were observed in *Crassostrea hongkongensis* (Lam & B. Morton, 2003) collected from estuaries in southern China enriched in industrial effluent releases [90]. The study showed that the shells of bivalves accumulated particularly high metal levels [94]. Fish are also highly sensitive to heavy metal pollution, making them suitable bioindicators for aquatic ecosystem monitoring because they readily metabolize, detoxify, and accumulate heavy metals within the body [95].

Beg et al. [96] confirmed that tilapia fish fingerlings *Oreochromis spilurus* (Günther, 1894) exhibited sensitivity to marine sediment elutriate suspensions in seawater, where their survival was reduced, and selected it as a suitable bioindicator for metal (Cd, Cr, Cu, Ni, Pb, Va, Zn) pollution in marine sediments. Other fish species, such as yellowfin seabream (*Acanthopagrus latus* (Houttuyn, 1782)) and tonguesole (*Cynoglossus* arel (Bloch and Schneider, 1801)), are bioindicators of metal pollution in marine sediments, which demonstrate oxidative impacts in tissues [97].

## 4. Case of Kuwait Bay (Northwestern Arabian Gulf) and Metal Pollution

The differences in the pattern of metal occurrence in various organs of *P. pelagicus* and the significant increase of Cu and Zn concentrations are presumably associated with the 1991 Gulf War oil spill that affected Kuwait’s marine environment [98]. This illustrates the ability of decapods to accumulate metals to detectable levels [98]. Male blue crabs *pelagicus* [98,99,100] are burrowing decapod crustaceans that occur abundantly in sandy and sandy-muddy sediments in shallow waters. This study aimed to examine Cd, Cu, Pb, and Zn field concentrations in the sediment, water, and selected tissues (gills, hepatopancreas, and carapace) of *P. pelagicus* in the coastal areas of Shuwaikh Port, Shuaiba Port, and Al-Khiran, Kuwait.

**Figure 6:**
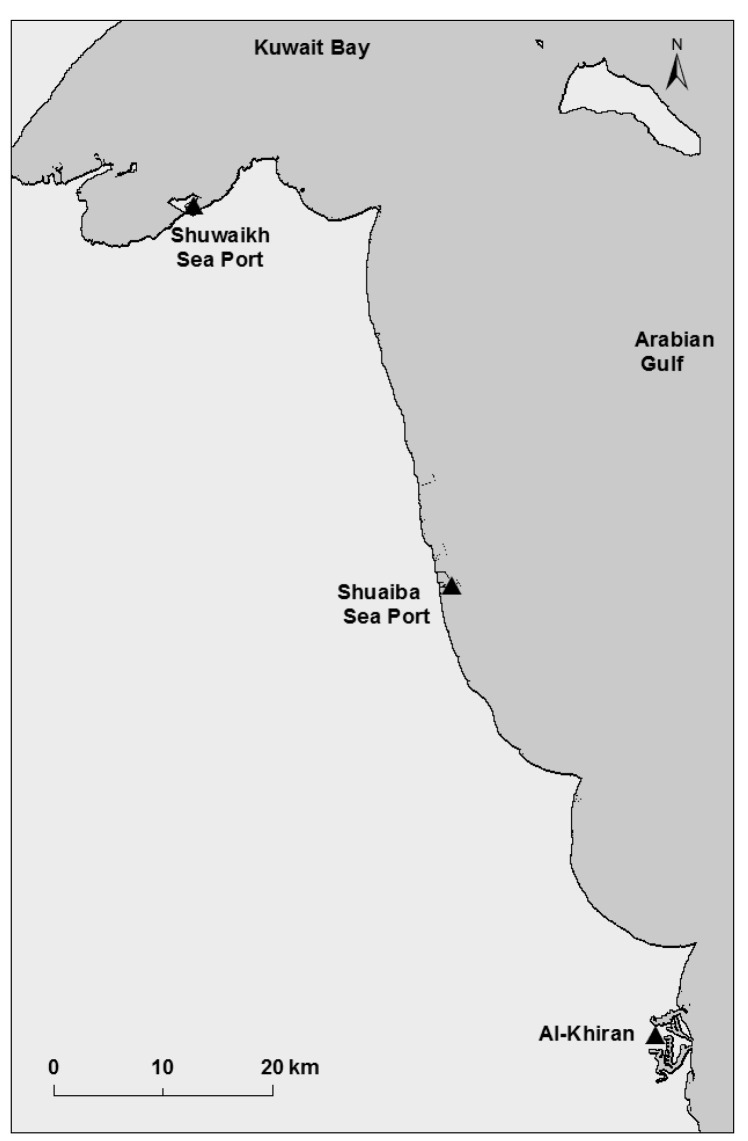
The three sampling locations in Kuwait waters: Shuwaikh Sea Port; Shuaiba Sea Port; and Al-Khiran.

Samples (crabs, sediment, and water) were collected from three geographic locations in Kuwait: Shuwaikh Port, Shuaiba Port, and Al-Khiran (Figure 6). *P. pelagicus specimens* were collected at about 1 m (i.e., the average depth of crab burrow), using an acid-prewashed hand shovel and plastic bags packed in acid-prewashed plastic bags, preserved, and transported to the laboratory for analysis [98]. The samples were washed with double-distilled deionized water to remove debris. Crabs were stored in a freezer at −20 °C before trace metal analysis. Water samples from the three locations were collected in plastic bottles, preserved by adding a few drops of concentrated nitric acid, and stored in a freezer for metal analysis. All equipment, glassware, and plastic containers were prewashed, soaked in Aristar-grade diluted nitric acid (10% *v*/*v*), and rinsed completely in double-distilled and deionized water before use to avoid pollution [98].

Crabs were dissected for their tissues (carapace, hepatopancreas, and gills) and dried in an oven to a constant weight at 650 °C for at least 72 h according to [98]. The samples were then ground in a porcelain mortar (particle size 250 μm), and an amount of 0.5 g of collective dried tissue samples was triplicated and digested in 20 mL concentrated nitric acid for 24 h. Afterward, the digested samples were blended with 10 mL of concentrated nitric and perchloric acid (4:1) and heated on a hot plate at 1200 °C until evaporation of the acid mixture and dryness. Residues were made up to 20 mL solution by adding 20 mL solution of Milli-Q^®^ water with 20% nitric acid and filtering with Whatman© filter paper. Heavy metal concentrations in organs were analyzed by Inductively Plasma Optical Emission Spectrometry (ICP-OES^®^, Perkin Elmer, Waltham, MA, USA). The sediments were oven-dried at 600 °C to a constant weight for at least 48 h [26]. The dried sediments consisted of fine particles, and the mud was ground into a powdery form using a ball grinder. Analyses were performed on the <63 μm fraction of the sediments, which were separated by sieving. One gram of homogenized sediment was digested with acid in triplicates, similar to the procedure used for tissue samples. Water samples were filtered with a 0.45-μm filter paper (Millipore Corporation, Burlington, MA, USA). An amount of 5 mL of water samples were digested in 10 mL of Aristar nitric acid, made up to 50 mL with deionized water [98]. The Quality assurance and quality control (QA/QC) of the analytical methods of metal detection in the crabs, sediments, and seawater were examined with oyster tissue (SRM 1566a), Peruvian sediment (SRM-4355), and seawater (QC 3163) certified reference materials to test for the analytical ability of Cd, Cu, Pb, and Zn [98]. The results of the QA/QC are displayed in Table 1.

One-way ANOVA and paired comparisons using post hoc Tukey’s test were conducted on the metal concentration between the three studied areas and between different crab tissues. Pearson’s correlation coefficient was tested to resolve the relationship between total metal concentrations in the crab tissues, sediments, and seawater using Xl-stat 2019 v. 21.2 software [101]. The data recorded in this study were submitted to a normalized Principal Component Analysis (PCA) [102].

### 4.1. Metal Concentrations in Crab Tissues

The mean concentrations of metals in the crab tissues from the Shuwaikh, Shuaiba, and Al-Khiran areas were turned out in the following order: Cu > Zn > Pb > Cd. Metal accumulation in crabs was higher in the carapace > gill > hepatopancreas. The highest metal accumulation was in crabs collected from Shuwaikh > Shuaiba > Al-Khiran. The results of metal concentrations in crab tissues varied according to the sampling stations (Table 2).

Cadmium (Cd) mean concentrations ± standard deviation (mean ± SD) were highest in the hepatopancreas> carapace > gills in the Shuwaikh Area, respectively (Table 2). Cd mean concentrations ± standard deviation were highest in the carapace > hepatopancreas> gills in Shuaiba Area, respectively. Cd (mean ± SD) concentrations ± standard deviation were highest in the hepatopancreas> carapace = gills in Al-Khiran Area, respectively (Table 2). Moreover, the ANOVA test showed that the Cd concentration differed significantly between the three studied areas (*p* < 0.001) (Table 2).

Copper (Cu) mean ± SD concentrations ± standard deviation were significantly higher in the carapace and gills than those in the hepatopancreas in the Shuwaikh Area (*p* < 0.001) (Table 2). Cu concentrations ± standard deviation were highest in the gills > carapace > hepatopancreas in the Shauiba Area, respectively (*p* < 0.001) (Table 2). Cu concentrations ± standard deviation were highest in the gills > hepatopancreas> carapace in Al-Khiran Area, respectively (*p* < 0.001) (Table 2). Cu concentration in different tissues differed significantly between the three studied areas (*p* < 0.001).

Lead (Pb) concentrations ± standard deviation were highest in the hepatopancreas> gills > carapace in the Shuwaikh Area, respectively (Table 2). Pb concentrations ± standard deviation were highest in the hepatopancreas the > gills > carapace in the Shuaiba Area, respectively (Table 2). Pb concentrations ± standard deviation were highest in the carapace > hepatopancreas > gills in the Al-Khiran Area, respectively (Table 2). Pb concentration in different tissues differed significantly between the three studied areas (*p* < 0.001).

Zinc (Zn) concentrations ± standard deviation were highest in the gills > carapace > hepatopancreas in the Shuwaikh Area, respectively (Table 2). Zn concentrations ± standard deviation were highest in the gills > carapace > hepatopancreas in the Shuaiba Area, respectively (Table 2). Zn concentrations ± standard deviation were highest in the carapace > gills > hepatopancreas in the Al-the Khairan Area (Table 2). Zn concentration in different tissues differed significantly between the three studied areas (*p* < 0.001).

Generally, the highest metal concentration determined was Cu (80.5 ± 1.49 μg/g) in *P. pelagicus* carapace sampled from the Shuwaikh Area. In opposition, the lowest metal concentration detected was Cd (0.16 ± 0.01 μg/g) in crab gills in the Shuaiba Area. Nonetheless, the concentration of Cd in the Shuaiba and Al-Khiran areas in hepatopancreas (0.16 ± 0.02 μg/g) was not significantly different (Tukey, *p* > 0.05) from that of gills in the same area (Table 2).

The Pearson’s Correlation coefficient linking the total heavy metals (Cd, Cu, Pb, and Zn) concentrations in the crab, sediment, and water measured in the Shuwaikh, Shuaiba, and Al-Khiran areas were significantly correlated (*p* < 0.05) (Table 3). Total metal in the tissue of crab from the three studied areas was positively correlated with metal in water (*p* < 0.001).

### 4.2. Metal Concentrations in Sediments

Heavy metal concentrations (Cd, Cu, Pb, and Zn) in sediments from Shuwaikh, Shuaiba, and Al-Khiran appear in the order; Zn > Cu > Pb > Cd (Table 4). Cd concentrations ± standard deviation were highest in Al-Khiran > Shauiba > Shuwaikh, respectively (Table 4). Cu concentrations ± standard deviation were highest in Al-Khiran > Shuwaikh > Shauiba, respectively. Pb concentrations ± standard deviation were highest in Al-Khiran > Shauiba > Shuwaikh, respectively. Zn concentrations ± standard deviation were highest in Al-Khiran > Shuwaikh > Shauiba, respectively (Table 4). The highest metal concentration detected in marine sediment was Zn (49.29 ± 1.69 μg/g) sampled from the Al-Khiran Area. However, the lowest metal concentration determined was Cd (2.63 ± 0.04 μg/g) in sediments from the Shuwaikh Area.

### 4.3. Metal Concentrations in Seawater

Cd concentrations ± standard deviation were highest in the waters sampled from Al-Khiran > Shauiba > Shuwaikh, respectively (Table 4). Cu concentrations ± standard deviation were highest in Shuwaikh > Shauiba > Al-Khiran, respectively. Pb concentrations ± standard deviation were highest in Shuwaikh > Al-Khiran > Shauiba, respectively. Zn concentrations ± standard deviation were highest in Al-Khiran > Shauiba > Shuwaikh, respectively (Table 4). The highest metal concentration detected in marine water was Zn (149.0 ± 10.28 μg/g) sampled from the Al-Khiran Area. In opposition, the lowest metal concentration determined in water was Cd (8.22 ± 2.32 μg/g) in water from the Shuwaikh Area. The mean concentrations of Cd and Cu were not significantly noticeable (*p* > 0.05) in water from the investigated areas (Table 4). The surrounding water quality measurements of the three study areas are displayed in Table 5.

### 4.4. Multivariate Analysis

**Figure 7:**
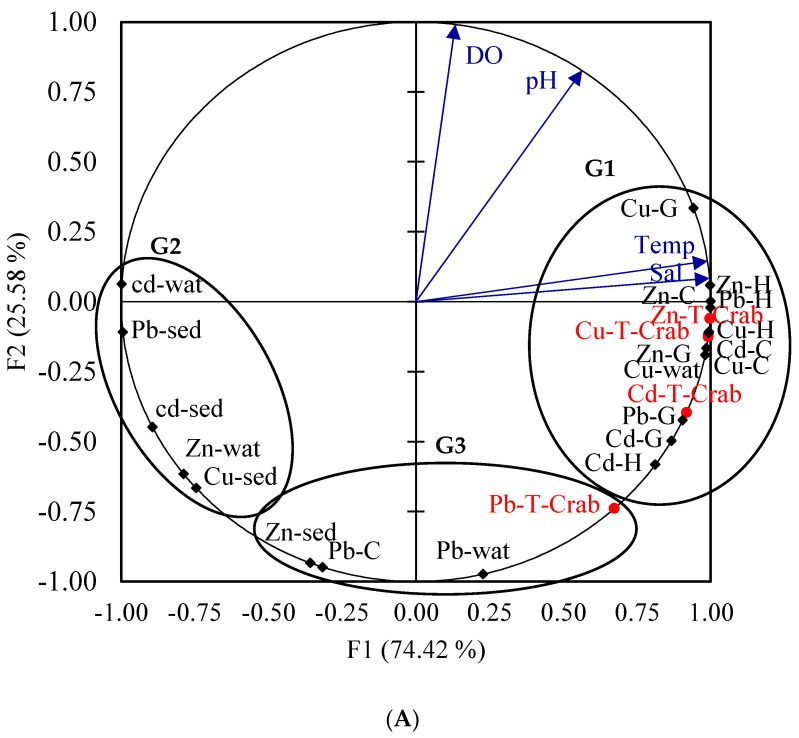

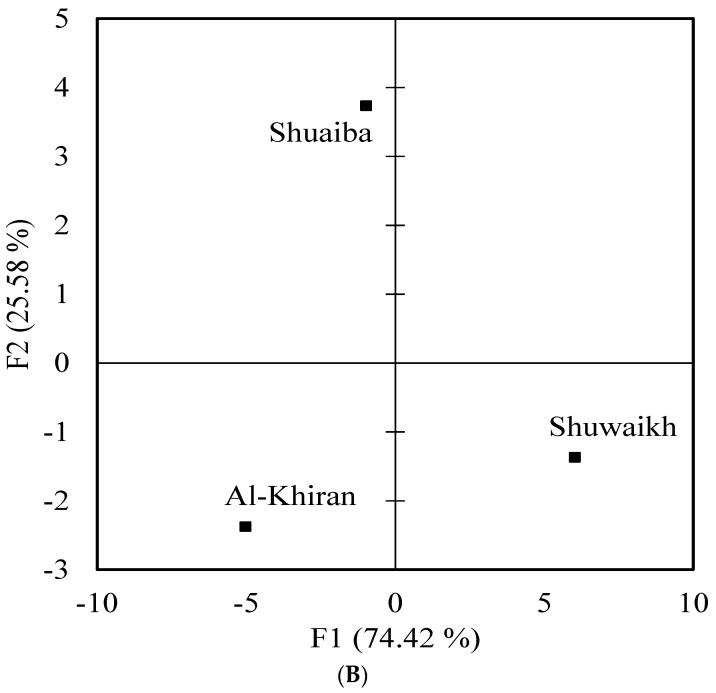
Principal component analysis (PCA): (**A**) Biplot of variables made on the metal amounts recorded in water, sediment, and different tissues of crabs and physical parameters of water. (**B**) Biplot of observations showing the distribution of the study areas: Shuwaikh, Shuaiba, and Al-Khiran (down). C: carapace, G: Gill, H: Hepatopancreas, Sed: sediment, Wat: water, T: total, Cu: copper, Pb: lead, Cd: cadmium, Zn: zinc.

Discrimination between the three studied areas was assessed by examining the projection of plots of the extracted factors on the factorial plane consisting of the statistically significant axis of the PCA analysis (Figure 7). The principal components analysis (PCA) showed 74.42% and 25.58% of the total variance in axis-1 (F1) and axis-2 (F2) (Figure 7). The plot of field observations showed a clear segregation between observations made in the studied areas: Al-Khiran, Shuaiba, and Shuwaikh (Figure 7B). The observations were grouped in the positive part of axis F1 together with zinc, copper, and cadmium metals accumulated in different parts of crabs, which were coupled with copper in water, salinity, and temperature of water (G1). However, the negative part of F1 negatively selected Cd and Zn in the water and Pb, Cd, and Cu in the sediment (G2). We assumed that the population of crabs in the Shuwaikh Area could be used as a bioindicator of copper in water. Axis F2 negatively selected the total lead in crabs, lead in carapace, lead in water, and zinc in sediment (G3). The latter group corresponds to the Al-Khiran Area, where crabs accumulate lead from the water column (Figure 7B).

## 5. Discussion

Heavy metal absorption levels in marine animals, especially the ability to accumulate in the different organs of crabs, are of particular interest due to the probable transfer and biomagnification along the food chain [100]. The present investigation indicated statistically significant inequality in organ (carapace, hepatopancreas, and gill) concentrations of metals (Cd, Cu, Pb, and Zn), as presumed. Significant differences in the tissue concentrations of Cd, Cu, Pb, and Zn have been reported in the mangrove crab *Sesarma mederi* (H. Milne Edwards, 1853) [103] and *Cardisoma armatum* (Herklots, 1851) [104]. Bioconcentrations of Cu and Zn (essential metals) in crabs from all study locations were higher than those of Cd and Pb (nonessential metals). In fact, the elevated concentrations of Zn and Cu might be due to their crucial role in maintaining physiological functions through various enzymatic activities [105]. Consequently, the uptake of these two essential micronutrients is higher than that of Cd and Pb, leading to a higher bioaccumulation of Zn and Cu in crabs. Raised concentrations of essential metals (such as Cu and Zn) in *P. pelagicus* exhibited in this study are an ordinary characteristic of marine crustaceans due to their potential to regulate these metal concentrations according to their physiological needs [106]. Concentrations of Cu were higher in the gills than in the hepatopancreas and carapace in Shuaiba and Al-Khiran. High concentrations of Cu in the gills could be a result of Cu being a component of hemocyanin, which is a respiratory pigment with its site of action in the gills [69]. Being nonessential metals, Cd and Pb accumulated more in the hepatopancreas, detoxification organs, than in the gills. Thus, the accumulation of nonessential metals in tissues induces detoxification mechanisms implying multiple proteins (such as metallothionein) [106]. In this investigation, the carapace of the crab *P. pelagicus* showed the highest significant levels of nonessential metal concentrations when compared to the gill and hepatopancreas. The carapace is the most important storage tissue, storing more metals as crabs grow [107]. Accordingly, the carapace is favored as the most convenient tissue compartment to sample when compelled by measure and time. The crab can eliminate the metals accumulated in the carapace during the molting period. The concentration and uptake of metals by crabs rely on environmental circumstances, as a surge in bioavailable metal concentrations results in high metal accumulation [108,109]. It is worth mentioning that the body concentration of the metal observed to be abnormally high for one species could be considered too low for another [110]. Accordingly, in a single taxon, metal concentration levels in crab tissues and the whole body vary substantially, even in the absence of anthropogenic metal pollution [98]. A metal frequently binds to proteins, for instance, in soluble metalliferous granules [110].

Particulate matter of sediments is composed of various mixed components and phases, which consist of organic material, carbonates, hydrous metal oxides, and crystalline minerals; thus, heavy metals could be bound to a particulate material via a range of processes [111,112]. Therefore, the dissemination of trace metals in individual phases clarifies their toxicity, mobility, and bioavailability [111,112]. The inverse relationship between the high heavy metal concentrations in sediments and noticeably low metal concentrations in the crab organs from the Al-Khiran Area compared to those from the Shuwaikh and Shuaiba Ports areas could point out the geological aspects. Consequently, they arise naturally in naturally high background levels of metals in the Al-Khiran Coastal Area, which could firmly influence the bioavailability of heavy metals, as the free ionic forms are consistently the most bioavailable [113,114]. Consequently, there is generally a strong negative relationship between the grain size of sediments and heavy metal concentrations [115,116]. Hence, the intensity of refinement of sediments investigated from Al-Khiran could also be accountable for the increased natural concentration of heavy metals. Although the Al-Khiran Coastal Area seems to have recovered largely from this anthropogenic event, the consequent effects of this event could have added to the inflated metal concentrations in the system.

Analysis revealed similar patterns for metal concentrations in both water and sediment samples, in which Zn > Cu > Pb > Cd. The three investigated study sites showed a mean concentration of the analyzed metals (Cd, Cu, Pb, and Zn) that was higher than the marine water quality guideline levels [117,118]. Metal concentrations in water from the Al-Khiran Coastal Area were not remarkably distinct from the other two investigated sites. The reason for the high concentrations of metals in the Al-Khiran Coastal Area could be attributed to high natural background metal concentrations. In addition, a possible explanation for high metal background concentrations is the 1991 Gulf War, which resulted in the deposition of high metal and the subsequent desorption of metals from the sediment compartment into the water column. It has been established that fluctuations in pH levels in seawater could eventually affect metal solubility. Alterations in seawater chemistry due to pH fluctuations can affect metal solubility, distribution, and speciation in the aqueous and solid phases [119]. Generally, the heavy metal load of marine sediments surpasses the one in seawater by considerable orders of magnitude [120], making sediment a crucial sink for metals. Despite this, a decrease in pH concentration can consequently increase both metal solubility and desorption from both sediments and organic ligands, ultimately leading to an extreme influx of heavy metals into the water column [121,122].

## 6. Conclusions

The outcome of this study indicates that the marine crab *P. pelagicus* could be used as a relevant biosentinel (quantitative bioindicator) due to its susceptibility to high accumulation of metals, notably in its carapace. Consequently, due to their deposit-feeding, submerging, and burrowing habits, they are prospective bioindicators for evaluating heavy metal pollutants in marine ecosystems where they are distributed extensively. The heavy metal extent in the sediments from the three investigated areas (Shuwaikh Port, Shuaiba Port, and Al-Khiran Coastal Area) was comparatively lower than the Australian sediment quality specification value, showing no negative ramifications to benthic organisms in these areas. The accumulation of Cd, Cu, Pb, and Zn in seawater from these investigated areas was above the water quality regulation mark. The results of this study will be beneficial in policy decision-making for regulatory authorities for better environmental management of crab habitats devastated by chemical pollution.

## Figures and Tables

**Table 1:** Validated and measured standards of heavy metal concentrations in certified reference material.

	Cadmium (Cd) µg/g	Copper (Cu) µg/g	Lead (Pb) µg/g	Zinc (Zn) µg/g
Water	Measured 8.94 ± 0.12	Measured 22.2 ± 0.96	Measured 0.51 ± 1.42	Measured 70.02 ± 0.38
	Certified 9.49 ± 0.15	Certified 22.7 ± 2.39	Certified 0.53 ± 0.29	Certified 60.81 ± 4.59
	% Recovery 96.8	% Recovery 112	% Recovery 109	% Recovery 108
Sediment	Measured 7.15 ± 1.87	Measured 107 ± 2.64	Measured 169 ± 3.91	Measured 911 ± 61.67
	Certified 5.63 ± 0.89	Certified 112 ± 20.41	Certified 192 ± 22.18	Certified 873 ± 179
	% Recovery 127	% Recovery 90.7	% Recovery 94	% Recovery 101
Crab	Measured 111 ± 22.9	Measured 176 ± 44.59	Measured 208 ± 29.6	Measured 49.22 ± 16.8
	Certified 112 ±1.96	Certified 186 ± 3.93	Certified 198 ± 2.17	Certified 53.04 ± 1.98
	% Recovery 106	% Recovery 96.9	% Recovery 107	% Recovery 92.8

**Table 2:** Heavy metal (mean ± SD) amounts in carapace, gills, hepatopancreas, and total crabs recorded in Shuwaikh, Shuaiba, and Al-Khiran study areas.

Heavy Metal	Cu					Zn				
Study Area	Shuwaikh	Shuaiba	Al-Khiran	F (d.f)	*p* Value	Shuwaikh	Shuaiba	Al-Khiran	F (d.f)	*p* Value
Carapace	80.5 ± 1.49 aA	25.2 ± 1.22 bA	16.9 ± 0.29 cA	2836.55 (8)	<0.0001 ***	25.9 ± 0.27 aAB	8.92 ± 0.31 bA	6.87 ± 0.19 cA	4787.89 (8)	<0.0001 ***
Gills	79.1 ± 2.99 aA	53.8 ± 5.01 bB	24.9 ± 1.01 cB	188.81 (8)	<0.0001 ***	29.1 ± 3.96 aB	14.2 ± 1.39 bB	6.82 ± 0.58 cA	64.59 (8)	<0.0001 ***
Hepatopancreas	68.6 ± 1.23 aB	22.6 ± 0.18 bA	14.9 ± 0.21 cA	4774.43 (8)	<0.0001 ***	18.9 ± 0.69 aA	6.92 ± 0.21 bA	4.74 ± 0.21 cB	927.11 (8)	<0.0001 ***
Total Crab	228.20 ± 5.71 aC	101.60 ± 6.41 bC	56.70 ± 1.51 cC	936.97 (8)	<0.0001 ***	73.9 ± 4.92 aC	30.04 ± 1.91 bC	18.43 ± 0.98 cC	267.33 (8)	<0.0001 ***
F (d.f)	1540.0 (11)	238.68 (11)	1315.63 (11)			185.46 (11)	230.12 (11)	337.48 (11)		
*p* values	<0.0001 ***	<0.0001 ***	<0.0001 ***			<0.0001 ***	<0.0001 ***	<0.0001 ***		
Heavy Metal	Pb					Cd				
Study area	Shuwaikh	Shuaiba	Al-Khiran	F (d.f)	*p* value	Shuwaikh	Shuaiba	Al-Khiran	F (d.f)	*p* value
Carapace	2.39 ± 0.11 aA	1.27 ± 0.19 bA	3.76 ± 0.21 cA	151.65 (8)	<0.0001 ***	0.39 ± 0.01 aA	0.24 ± 0.03 bA	0.19 ± 0.02 bA	69.64 (8)	<0.0001 ***
Gills	3.29 ± 0.29 aA	1.59 ± 0.21 bA	1.06 ± 0.02 cB	94.99 (8)	<0.0001 ***	0.28 ± 0.02 aB	0.16 ± 0.01 bA	0.19 ± 0.02 bA	39.00 (8)	0.000 ***
Hepatopancreas	4.69 ± 0.29 aB	1.60 ± 0.12 bA	1.76 ± 0.21 aC	191.01 (8)	<0.0001 ***	0.43 ± 0.02 aA	0.16 ± 0.02 bA	0.20 ± 0.02 bA	159.25 (8)	<0.0001 ***
Total Crab	10.37 ± 0.69 aC	4.46 ± 0.52 bB	6.58 ± 0.44 cD	85.82 (8)	<0.0001 ***	1.10 ± 0.05 aC	0.56 ± 0.06 bB	0.58 ± 0.06 bB	86.97 (8)	<0.0001 ***
F (d.f)	234.81 (11)	73.44 (11)	260.23 (11)			488.71 (11)	87.04 (11)	93.50 (11)		
*p* values	<0.0001 ***	<0.0001 ***	<0.0001 ***			<0.0001 ***	<0.0001 ***	<0.0001 ***		

F-value: between-groups mean square/within-groups mean square. d.f: degree of freedom. a, b, c, per row denote significant differences between study areas as tested with one-way ANOVA and paired comparisons using Tukey’s test (*p* < 0.05). A, B, C, D per column denote significant differences between samples of crabs as tested with one-way ANOVA and paired comparisons using Tukey’s test (*** *p* < 0.01).

**Table 3:** Pearson’s Correlation Coefficient (R) between total heavy metal concentrations in the crab tissue, sediment (Sed), and water (wat) measured in the Shuwaikh (Shkh), Shuaiba (Shu), and Al-Khiran (Kh) areas.

Variables	Wat-Shkh	Sed-Shkh	Tissue-Shkh	Wat-Shu	Sed-Shu	Tissue-Shu	Wat-Kh	Sed-Kh	Tissue-Kh
wat-Shkh	1								
Sed-Shkh	0.248	1							
Tissue-Shkh	0.913 ***	0.313	1						
wat-shu	0.981 ***	0.318	0.971 ***	1					
Sed-Shu	0.349	0.612 *	0.186	0.331	1				
Tissue-shu	0.919 ***	0.286	0.999 ***	0.972 ***	0.171	1			
wat-Kh	0.578 *	0.925 ***	0.622 *	0.641 *	0.628 *	0.604 *	1		
Sed-Kh	0.441	0.949 ***	0.392	0.461	0.722 **	0.368	0.933 ***	1	
Tissu-Kh	0.938 ***	0.303	0.998 ***	0.983 ***	0.207	0.997 ***	0.618 *	0.403	1

* *p* < 0.05; ** *p* < 0.01, *** *p* < 0.001.

**Table 4:** Heavy metal (Cd, Cu, Pb, and Zn) accumulations in the sediments and water from Shuwaikh, Shuaiba, and Al-Khiran. (Mean value (μg/g) ± SD).

Compartiment	Sediment					Water				
Study Area	Shuwaikh	Shuaiba	Al-Khiran	F(d.f)	*p* Values	Shuwaikh	Shuaiba	Al-Khiran	F(d.f)	*p* Values
Cd	2.63 ± 0.04 a	3.29 ± 0.25 b	6.72 ± 0.12 c	552.77 (8)	<0.0001 ***	2.63 ± 0.04 a	3.29 ± 0.25 a	6.72 ± 0.12 a	0.87 (8)	0.467
Cu	15.95 ± 0.84 a	15.23 ± 0.27 a	33.12 ± 0.44	949.56 (8)	<0.0001 ***	1.95 ± 0.84 a	15.23 ± 0.27 a	33.12 ± 0.44 a	1.93 (8)	0.226
Pb	11.42 ± 0.43	19.42 ± 1.79	30.23 ± 1.27	160.34 (8)	<0.0001 ***	11.42 ± 0.43 a	19.42 ± 1.79 b	30.23 ± 1.27 a	14.69 (8)	0.005 **
Zn	33.12 ± 0.79 ab	22.02 ± 16.14 a	49.29 ± 1.69 b	6.41 (8)	0.032 *	33.12 ± 0.79 a	22.02 ± 16.14 a	49.29 ± 1.69 a	126.68 (8)	<0.0001 ***

F-value: between-groups mean square/within-groups mean square. d.f: degree of freedom. a, b, c, per row denote significant differences between study area for each heavy metal as tested with one-way ANOVA and paired comparisons using Tukey’s test (* *p* < 0.05; ** *p* < 0.01, *** *p* < 0.001).

**Table 5:** Background physicochemical properties of the water in Shuwaikh, Shuaiba, and Al-Khiran (±SD). *n* = 45.

Areas Water Parameters	Shuwaikh	Shuaiba	Al-Khiran
Salinity (psu)	40.9 ± 0.98	40.2 ± 0.26	39.8 ± 0.83
Dissolved oxygen (mg/L)	4.35 ± 0.56	4.95 ± 0.69	4.12 ± 0.87
Temperature (°C)	23.4 ± 0.67	22.2 ± 0.97	21.3 ± 0.54
pH	8.06 ± 0.18	8.24 ± 0.94	7.65 ± 0.65
